# Risky Play Is Not a Dirty Word: A Tool to Measure Benefit–Risk in Outdoor Playgrounds and Educational Settings

**DOI:** 10.3390/ijerph22060940

**Published:** 2025-06-16

**Authors:** David Eager, Tonia Gray, Helen Little, Fiona Robbé, Lisa N. Sharwood

**Affiliations:** 1 Faculty of Engineering and Information Technology, University of Technology Sydney, P.O. Box 123, Broadway, NSW 2007, Australia; 2 School of Education, Western Sydney University, Locked Bag 1797, Penrith, NSW 2751, Australia; t.gray@westernsydney.edu.au; 3 World Leisure Centre for Excellence, Western Sydney University, Locked Bag 1797, Penrith, NSW 2751, Australia; 4 Macquarie School of Education, Macquarie University, Wallumattagal Campus, Macquarie Park, NSW 2109, Australia; helen.little@mq.edu.au; 5 Architects of Arcadia, 108 Arcadia Road, Arcadia, NSW 2159, Australia; fiona@architectsofarcadia.com.au; 6 School of Population Health, Faculty of Medicine and Health, University of New South Wales, Sydney, NSW 2032, Australia; l.sharwood@unsw.edu.au

**Keywords:** antisocial behaviour, benefit risk, challenge and adventure, childhood development, educational settings, ISO 4980:2023, learning environment, outdoor education, playgrounds, recreational risk, risky play, well-being

## Abstract

Challenge, adventure, and risky play have repeatedly been found to be learning environments that positively shape childhood well-being and development. Extant evidence-based research conveys the physical, cognitive, and socio-emotional growth associated with risky play provision. However, understanding the interplay of risky play, injury, and safety is more nuanced and complex. The goal of this paper is to introduce a tool which allows educators, parents, health practitioners, urban planners, playground designers, certifiers, manufacturers, and inspectors to estimate both the benefit and risk of outdoor play and learning settings, such as playgrounds, adventure parks, or risk-taking activities. One of the key challenges associated with societal risk appetite or risk tolerance has been the inability to quantify the inherent benefits of risk taking in playgrounds and educational settings. Historically, the assessment of ‘benefit–risk’ has been dominated by a zero tolerance of incidents, whether in the workplace or road safety settings. Against this backdrop, if playgrounds and outdoor learning settings are boring, children will go elsewhere to seek thrills and adventure, which may often be manifested in antisocial behaviour. In 2023, ‘benefit–risk’ was formally recognised in the area of sport and recreation by the International Organisation for Standardisation, when it published the ISO 4980:2023 benefit–risk assessment for sport and recreational facilities, activities, and equipment. ISO 4980:2023 is a departure from the traditional view of risk management, in that it presents a perspective of risk which is not limited to framing risk as negative, but rather balances the calculation of positive benefits as well as the risks associated with the activity. Correspondingly, hazardous situations which have zero benefit should be eliminated or mitigated. The tool introduced in this paper offers playground inspectors and educators the ability to measure and assess both the benefit and risk of outdoor playgrounds and educational settings where children play, learn, and flourish.

## 1. Introduction

The publication of the international standard ISO 4980:2023 benefit–risk assessment for sport and recreational facilities, activities, and equipment [1] (ISO 4980:2023) has changed the way risk assessments are now conducted and assessed within sport and recreational industries around the world. ISO 4980:2023 introduced the concept of benefit–risk—a radical departure from the historical ways of assessing recreational settings to align them with pedagogical outcomes. ISO 4980:2023 defines benefit–risk as:

“*The concept which acknowledges that in sports and recreation there is an inevitable and inherent trade-off between the benefits of a sport or recreational activity, and some of the risks which they can pose.*”

The provision of play serves a fundamental role in both formal and informal learning landscapes. The distinction between ‘formal’ and ‘informal play’, however, is often contentious. By its very nature, play is the incubator for ‘learning by doing’ or experiential learning [2,3,4]. Informal play is inherently self-driven by curiosity and wonder. When a child naturally engages in play by exploring their surroundings or experimenting with objects, they develop a deeper understanding of the world. Alternatively, formal learning landscapes such as preschools and early childhood education centres are powerful vehicles for educators to enrich the educative process through the provision of play opportunities.

The requirement of play provision in the formative years of childhood is recognised in the United Nations Convention on the Rights of the Child [5]. Learning environments where children are encouraged to engage in free and unstructured play, are also cultivating a myriad of social, emotional, and executive functioning skills which are critical for lifelong success [6,7].

Across the world, the premise that there is an inherent benefit associated with risk has been widely acknowledged within educational settings and associated allied health fields [3,8,9,10,11,12,13,14]. Risk taking and experimentation are an integral part of childhood as children seek to understand and navigate their environment [4,15]. The benefits associated with risk taking in childhood have been embraced by multidisciplinary fields such as educational policy makers, playground providers, landscape architects, and playground designers [9,16,17,18,19,20,21,22,23,24,25,26,27,28,29,30]. More recently, evidence-based research has emerged that supports the myriad of benefits associated with well-managed risk in childhood play spaces [2,11,31,32,33,34,35,36,37,38]. Against this backdrop, the benefits that come from risk taking include educational, psychosocial, cognitive, emotional, and physical benefits [28,39,40].

Play experts have long been concerned that play spaces do not provide children with adequate exposure to sufficient risk. In 1995, Heseltine stated [41]:

“*We have made the playground so monumentally boring that any self-respecting child will go somewhere else to play, somewhere more interesting, and usually more dangerous.*”

Heseltine essentially criticised contemporary society of sanitising the risk from playgrounds. He postulated that removing appropriate levels of children’s exposure to risk would be detrimental to the health and well-being of society, particularly children.

The benefit–risk concept also has its roots and early adoption in the design and operation of outdoor playgrounds in the UK. In 2002, the Play Safety Forum published a position statement titled ‘Managing risk in play provision: A position statement’ [42] (Play Safety Forum). The Summary Statement within this document stated:

“*Children need and want to take risks when they play. Play provision aims to respond to these needs and wishes by offering children stimulating, challenging environments for exploring and developing their abilities. In doing this, play provision aims to manage the level of risk so that children are not exposed to unacceptable risks of death or serious injury.*”

Two years later, Standards Australia embraced the important role that play provides children when they published AS 4685.1:2004 [43] (AS 4685.1:2004). The Forward to AS 4685.1:2004 states:

*“The primary aim of a playground should be to stimulate a child’s imagination, provide excitement and adventure in safe surroundings, and allow scope for children to develop their own ideas of play. Ideally playgrounds should encourage development of motor skills and present users with manageable challenges to develop physical skills and to find and test their limits. In order to provide these challenges, a balance must be found between risk and safety. Professional advice should be sought, and children should be involved in planning, to ensure that the playground satisfies children’s ideas of play.”*

Four years later, the European playground standard reinforced the importance of childhood play provision when they published EN 1176-1:2008 [44] (EN 1176-1:2008). The Introduction to EN 1176-1:2008 states:

*“Risk-taking is an essential feature of play provision and of all environments in which children legitimately spend time playing. Play provision aims to offer children the chance to encounter acceptable risks as part of a stimulating, challenging and controlled learning environment. Play provision should aim at managing the balance between the need to offer risk and the need to keep children safe from serious harm…*

*Respecting the characteristics of children’s play and the way children benefit from playing on the playground with regard to development; children need to learn to cope with risk. This may lead to bumps and bruises and even occasionally a broken limb. The aim of this standard is first and foremost to prevent accidents with a disabling or fatal consequence, and secondly to lessen serious consequences caused by the occasional mishap that inevitably will occur in children’s pursuit of expanding their level of competence, be it socially, intellectually or physically.”*

With the publication of AS 4685.0:2017 Playground equipment and surfacing Part 0: Development, installation, inspection, maintenance and operation [45] (AS 4685.0:2017), Australia was the first country to incorporate the benefits of play into the body of a national standard and make these a requirement. AS 4685.0:2017 requires playground operators (and their risk assessors) when assessing the risk associated with any particular playground, to also take into account the following factors: (1) the context of the playground; (2) its purpose; (3) its likely users; and (4) the need for benefit assessment procedures instead of standard risk removal [45]. The introduction to this standard states:

*“Play is a significant aspect of children’s lives through which they develop and demonstrate knowledge, skills, concepts and dispositions. It is an important context for all aspects of children’s learning and development including problem-solving, social-emotional development, the acquisition and mastery of physical skills, self-awareness, imaginative abilities, exploration of natural environments, and relaxation. Consideration of play provision should not be limited to formally-equipped areas but also extend to natural elements. Provision should also be made to cater for the needs and interests of users of all abilities.*

*It is important to recognise the value to children of engaging with nature. The incorporation of natural materials as design elements in a playground can add significant play, aesthetic and environmental value.*

*Natural elements incorporated into playgrounds are inherently diverse and open-ended, and many offer the benefit that children can manipulate them for their own play purposes. Such nature-based play environments can help build creativity, imagination and problem-solving skills. Access to nature has been shown to improve children’s psychological well-being and encourage stewardship of the environment.*

*It should be recognised that risk taking is an essential feature of play provision and of all environments in which children legitimately spend time playing. Play provision aims to offer children the chance to encounter acceptable risks as part of a stimulating and challenging learning environment. Play provision should aim at managing the balance between the need to offer risk and the need to keep children safe from serious harm.”*

The publication of AS 4685.0:2017 was a significant step on the journey which led to the publication of ISO 4980:2023 [1].

ISO 4980:2023 is a landmark development and significant refinement because it challenges, on the international stage, the long-accepted view that risk can only be negative in the sport and recreational space. The goal of this paper is to provide examples of the application of benefit–risk assessment (BRA) specific to outdoor playgrounds and educational settings.

## 2. Methodology

This manuscript uses the benefit descriptors presented within ISO 4980:2023 to develop a benefit–risk tool building on long-accepted risk assessment methods, such as the likelihood/consequence matrix. The likelihood/consequence matrix presented within IEC 31010:2019 Risk management—Risk assessment techniques (IEC 31010:2019) [46] was employed for the risk assessment. Risks are listed individually as separate events and any interdependence flagged. IEC 31010:2019 states:

*“Risks where consequences are positive can be recorded in the same document as those where consequences are negative or separately. Opportunities (which are circumstances or ideas that could be exploited rather than chance events) are generally recorded separately and analysed in a way that takes account of costs, benefits and any potential negative consequences. This can sometimes be referred to as a value and opportunities register.”*

**Figure 1:**
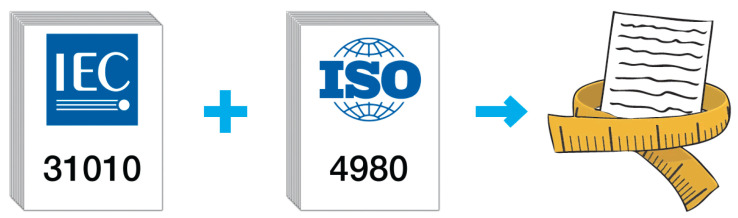
IEC 31010:2019 together with ISO 4980:2023 allowed the development of a tool for the measurement of benefit–risk within outdoor playgrounds and educational settings.

ISO 4980:2023 [1] takes the concept of separating positive risk to a higher level by defining these positive risks as benefits. In this paper, the authors employ a long-established and well-respected risk matrix methodology. A risk matrix provides a visual representation of the overall risk level, enabling better decision making and resource allocation. The methodology, in essence, plots the risk onto a grid with the likelihood of an accident or mishap on one axis, and the consequence on the other axis. This paper takes the likelihood/consequence matrix presented within IEC 31010:2019 [46] and uses the concepts contained within ISO 4980:2023 to develop a tool for the measurement of benefit–risk within outdoor playgrounds and educational settings. Figure 1 is a sketch illustrating the process which allows benefit–risk to be quantified.

A benefit/likelihood matrix is proposed for the benefit assessment using identical descriptors for the quantification of likelihood as the consequence/likelihood matrix. The use of identical likelihood descriptors enables the benefit assessment and the risk assessment to be directly compared and evaluated. Several benefit–risk examples of the application of this international standard in the context of children’s playgrounds are presented. Hazardous situations which have zero benefit are a special case and ideally should be eliminated, or mitigated if they cannot be eliminated. An example to illustrate a hazardous situation is also presented.

## 3. Benefit–Risk Assessment

**Figure 2:**
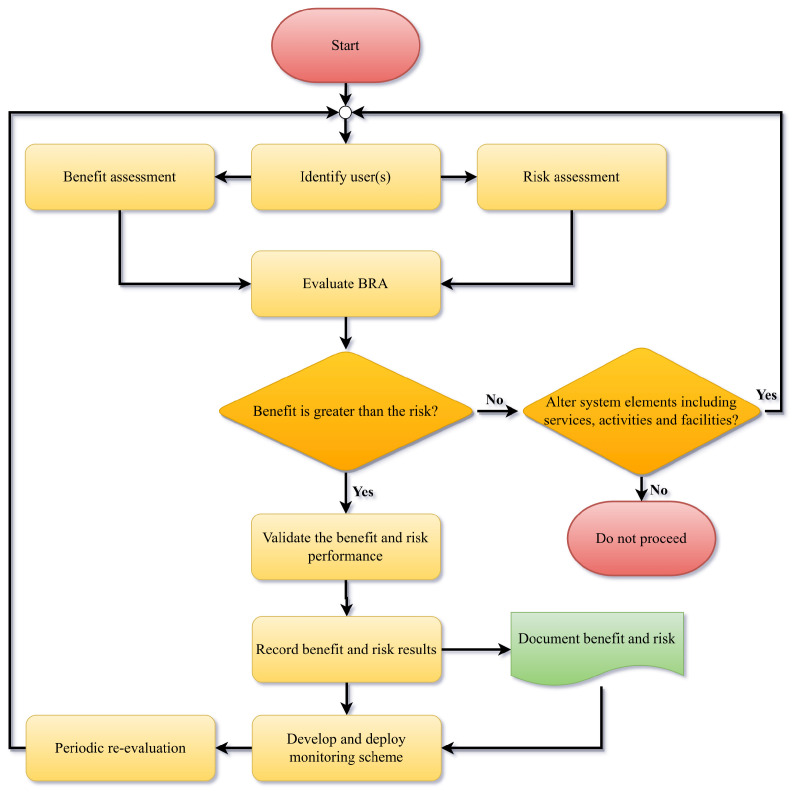
BRA flowchart depicting the evaluation of the benefit assessment and the risk assessment [38].

In 2024, Eager [38] published the BRA process in a paper titled *Benefit–Risk Assessment in Sport and Recreation: Historical Development and Review of AS ISO 4980:2023*. The BRA process requires the outdoor playground owner/ operator to conduct both a benefit assessment and a risk assessment. Figure 2 is a flowchart of the BRA process.

The discussion that follows provides the rationale for a tool which the authors of this paper have developed to assist playground inspectors conducting a BRA.

### 3.1. Establishing the Context

When conducting any risk assessment, the first step is to establish the context. Conducting a BRA is no different; the context must first be established. The context here is children playing in an outdoor playground.

### 3.2. Identification of User

Following the establishment of the context, the next step is to determine the expected users of the activity, product, service, or equipment.

### 3.3. Risk Assessment

**Figure 3:**
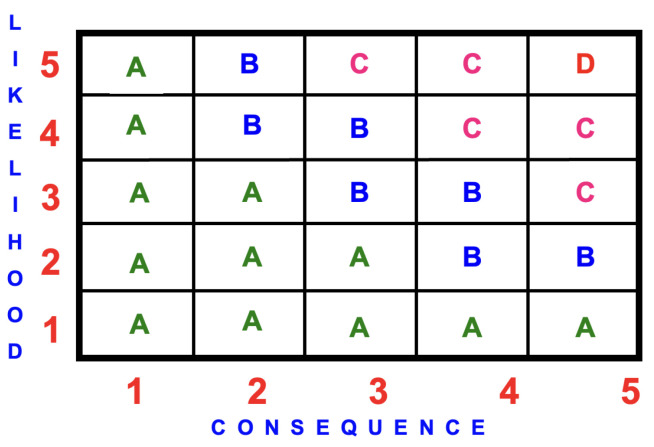
Example of a 5×5 consequence/likelihood matrix depicting the four risk scores, namely: (A) Low 1–7; (B) Medium >7-12; (C) High >12–20; and (D) Highest >20.

In the assessment of children’s outdoor playgrounds, the most usual assessment technique is the likelihood/consequence matrix, commonly known as a *risk severity matrix* or simply a *risk matrix* (consequence/likelihood matrix). This technique is derived from the likelihood/consequence matrix presented within IEC 31010:2019 [46]. The results are presented graphically with the consequences on the x-axis and likelihood on the y-axis. Figure 3 is an example of a consequence/likelihood matrix.

The consequence/likelihood matrix is currently used widely in the outdoor playground industry to communicate a common quantifiable understanding of risk exposure to other parties such as the playground owner/operator and the playground insurer. The way the playground inspector quantifies the risk likelihood should align with the playground owner/operator’s risk appetite.

For each identified risk, its likelihood and consequences are assessed and estimated using agreed descriptors. Customised ratings for consequence and likelihood are defined for the axes of the matrix. The ranking can have any range. Commonly used ranges are 3, 4, 5, 7, and 10. The rankings can be qualitative, semi-quantitative, or quantitative. When numerical descriptions are used to define the ranking, they should be consistent with available data and, preferably, evidence-based.

The following is an example of likelihood ratings (probability) and their associated descriptor for an incident occurring within a children’s playground using a scale of 5:

1 = Rare (highly unlikely event);
2 = Unlikely (conceivable event);
3 = Possible (could occur event);
4 = Likely (almost certain event);
5 = Certain (will occur event).

The consequences rating should be directly connected to the objectives of the playground owner/operator, and should extend from the lowest consequence of interest to the maximum credible consequence.

The following is an example of the potential consequences of an injury occurring within the playground being assessed using a scale of 5:

1 = Little or no injury;
2 = Minor injury requiring first aid;
3 = Moderate injury requiring medical assessment;
4 = Serious injury with long-term consequences;
5 = Death or major disability.

The risk score is calculated by multiplying the likelihood rating by the potential consequence rating to quantify the risk event according to the following scoring system, namely (see Figure 3):

(A) Low risk =1 to 7;
(B) Medium risk >7 to 12;
(C) High risk >12 to 20;
(D) Highest risk >20 .

### 3.4. Benefit Assessment

**Figure 4:**
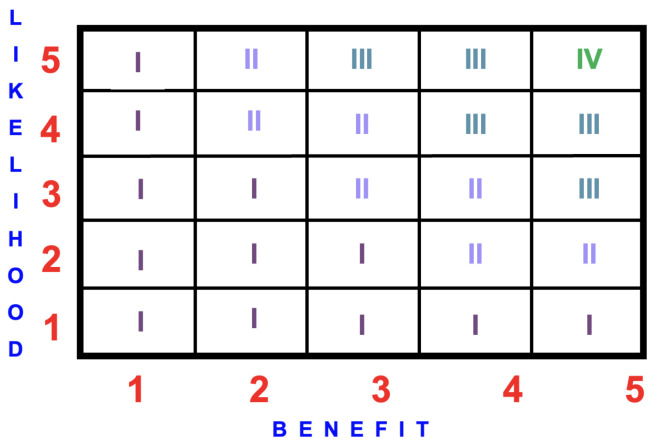
Example of a 5×5 benefit/likelihood matrix depicting the four benefit scores, namely: (I) Low 1–7; (II) Medium >7–12; (III) High >12–20; and (IV) Highest >20.

For the BRA to work correctly, the benefit assessment must use the same likelihood scale and descriptors as the risk assessment. It must also use the same benefit scale as the consequence scale. Figure 4 is an example of a benefit/likelihood matrix.

The following is an example of the benefit assessment which is compatible with the risk assessment previously presented within this paper.

Using the same rating as was used for the risk assessment, the likelihood of a benefit will be:

1 = Rare (highly unlikely event);
2 = Unlikely (conceivable event);
3 = Possible (could occur event);
4 = Likely (almost certain event);
5 = Certain (will occur event).

The five benefit descriptors in ISO 4980:2023 [1] provide a mechanism to semi-quantitatively rank the benefit of a sports and recreational facility, activity, and equipment. These descriptors are as follows:

1 = A momentary benefit such as joy in the activity.
2 = Short-term benefit such as having learned a new skill or learning skills faster and meeting and making new acquaintances.
3 = Medium-term benefit such as gaining proficiency that opens new opportunities and beginnings of a benefit feedback loop.
4 = Permanent life-style improvements that lead to better physical, social, and mental health have influence on future engagements and activities that are a further benefit to the user and are likely to contribute to the permanence of the benefit-feedback loop. This can also encourage and reinforce the user’s engagement in greater challenges.
5 = Benefits that go beyond the individual to engage others and potentially benefit society include reduction in suicide rates because of reduction of depression, resulting in, e.g., lower health care costs.

The benefit score is calculated by multiplying the likelihood rating by the benefit rating to quantify the benefit event according to the following scoring system, namely (see Figure 4):

(I) Low benefit =1 to 7;
(II) Medium benefit >7 to 12;
(III) High benefit >12 to 20;
(IV) Highest benefit >20.

## 4. Discussion

In 2024, the Canadian Paediatric Society published a position statement on healthy childhood development through outdoor risky play, in which they stated [15]:

*“Opportunities to engage in outdoor free play- and risky play in particular-have declined significantly in recent years, in part because safety measures have sought to prevent all play-related injuries rather than focusing on serious and fatal injuries. Risky play is defined by thrilling and exciting forms of free play that involve uncertainty of outcome and a possibility of physical injury. Proponents of risky play differentiate ‘risk’ from ‘hazard’ and seek to reframe perceived risk as an opportunity for situational evaluation and personal development. This statement weighs the burden of play-related injuries alongside the evidence in favour of risky play, including its benefits, risks, and nuances, which can vary depending on a child’s developmental stage, ability, and social and medical context. Approaches are offered to promote open, constructive discussions with families and organisations. Paediatricians are encouraged to think of outdoor risky play as one way to help prevent and manage common health problems such as obesity, anxiety, and behavioural issues.”*

This statement, in addition to other developments, has paved the way for groundbreaking discourse to emerge around the world between the interplay of risk literacy, risk tolerance, and risk appetite, in parallel with the cognitive and developmental benefits. Against this backdrop, the dialogue surrounding the benefits of risk is gaining visibility within a myriad of sectors, whether they be the educational sector, risk policy makers, or urban planners, for example [47].

**Figure 5:**
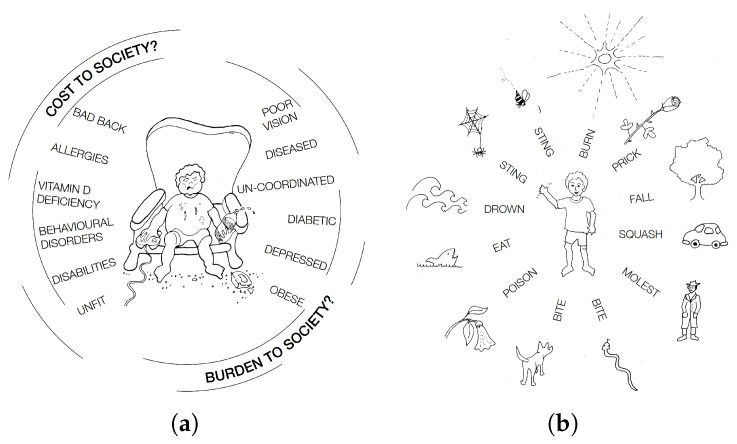
(**a**) Cost and burden to society. (**b**) Fear of nature.

The lack of appropriate risk can have negative consequences. For example, when there is a paucity of risk in playgrounds, children are less likely to be active and play, and this in turn will contribute to the burgeoning concern over children’s health and well-being [48]. In addition, children do not incrementally gain valuable risk assessment skills that are necessary to modern life. This is further compounded by parents, carers, and educators being fearful that contact with the Great Outdoors is risky in its own right and that this perhaps is the greatest risk of all. These inherent fears need to be balanced with the opposing argument of why playing outside is so valuable, if not essential, to a healthy childhood Figure 5 depicts: (a) the cost and burden to society and (b) the fear of nature.

Our discussion applies the concepts developed in the previous section to a number of commonly encountered situations within the context of outdoor play activities and outdoor learning environments such as playgrounds. Notably, before the publication of ISO 4980:2023 [1], only a risk assessment (see Figure 3) would have been conducted. The following eight examples will be used to illustrate how the benefit–risk measurement tool we propose can be applied in outdoor playgrounds and educational settings:

1. Rough and tumble play in a supervised early childhood setting;
2. Children participating in a nature play activity;
3. A child using a swing in an outdoor playground or school setting;
4. A child riding a flying fox in an outdoor playground;
5. A child on a rocking device in an outdoor playground (spring animal);
6. A group of children climbing up and playing on a 10 m high 3D spatial net;
7. Giant tube slide being used inappropriately and appropriately;
8. Broken glass bottle in the playground or school setting.

The case studies which follow were developed from several sources, including the following:

- The authors’ combined lived experience as life-long educators of children and adolescents;
- Educators and assessors of outdoor playgrounds and supervised early childhood settings;
- Inspectors and maintenance staff of outdoor playgrounds and supervised early childhood settings;
- Landscape architects and engineers in the field;
- Experts on Australian and International Standards technical committees.

The benefit criteria selected are universally accepted as the key benefits of play provision, i.e., globally, there is resounding recognition from teachers and educators that play enhances creativity, problem solving, coordination, proprioception, balance, and agility, gross motor skill development, physical activity, and building confidence [49].

### 4.1. Rough and Tumble Play in a Supervised Early Childhood Setting (R&T)

As a category of risky play, rough and tumble play is a form of fun, socially interactive, physical play characterised by behaviours such as wrestling, play fighting (using objects such as sticks or pool noodles), jumping, tumbling, and chasing, and include protect/rescue games, and super-hero play [50,51]. While it mimics behaviours that might be interpreted as aggressive, it is symbolic of aggression and players do not intend to hurt their play partners, as evidenced by the expression of a cheerful play-face, and shared understanding of the ‘rules’ of the game [52].

This example illustrates an everyday learning environment activity for preschool children which offers them a range of benefits for development and learning [53] including:

- Problem solving, cooperation, turn-taking, negotiation, and conflict resolution;
- Imagination and perspective-taking through role play;
- Communication skills;
- Physical skills including fine and gross motor skills, balance, agility, coordination, and strength;
- Moderate to vigorous physical activity, endurance, and fitness, associated with reduced obesity;
- Self-awareness, emotion regulation, compassion, empathy, and respect for others;
- Self-confidence, sense of belonging, and friendship formation.

#### 4.1.1. Risk Matrix

The magnitude of the likelihood that an injury occurs when a child is engaged in R&T play will depend largely on the form of play and the location. Are children playing on grass, hard-top surfaces, or surfaces with impact attenuating properties? Does the play involve the use of objects such as sticks? Are there trip hazards in the play environment? Depending on these variables, the likelihood of an injury occurring from a group of children engaged in R&T play in a supervised early childhood setting ranges from rare to possible. Incidents which can occur include trips and falls, children running into an object and/or other children, and scratches, bruises, or eye injuries associated with sharp objects used in play fighting. Where the use of sticks is permitted, rules related to ensuring they are only used to strike body areas lower than waist height should be implemented. Alternatively, the use of other objects such as pool noodles would present less of a hazard.

Likelihood =3.0 (possible).

Depending on the form of R&T play, the possibility of the consequence of an injury with a likelihood of 3.0 occurring would, in most scenarios, be a minor injury requiring first aid.

Consequence =2.0 (minor injury requiring first aid).

The risk score is 3.0×2.0=6.0 (low).

In instances where children are engaged in play fighting using sticks, the consequence of an injury with a likelihood of 3.0 occurring would be a moderate injury requiring medical assessment.

#### 4.1.2. Benefit Matrix

There are a number of different combinations of likelihood and benefit for this example. Five combinations for R&T play will be discussed.

Scenario 1
  A momentary benefit such as the elation or joy in the activity.
  The likelihood of this benefit occurring is ‘certain’.

The benefit score for Scenario 1 is 5.0×1.0=5.0 (low).

Scenario 2
  Short-term benefit such as having acquired a new skill or learning skills faster and building stronger friendships.
  The likelihood of this benefit occurring is ‘certain’.

The benefit score for Scenario 2 is 5.0×2.0=10.0 (medium).

Scenario 3
  Medium-term benefit relates to the beginnings of a benefit feedback loop resulting from children learning self-control, boundaries, and understanding of their own and others’ abilities and feelings.
  The likelihood of this benefit occurring is ‘likely’.

The benefit score for Scenario 3 is 4.0×3.0=12.0 (medium).

Scenario 4
  Lifelong benefits which lead to enhancing physical, social, and mental health and well-being outcomes inevitably have a positive influence on children’s further engagement in R&T play. Positive feedback children receive from play partners reinforces the benefits resulting in likely permanence within the benefit feedback loop. Engagement in R&T play intrinsically encourages children to seek this form of play in other contexts and other social groups.

The likelihood of this benefit occurring is ‘likely’.

The benefit score for Scenario 4 is 4.0×4.0=16.0 (high).

Scenario 5
  Benefits that go beyond the individual to engage more broadly with others to potentially benefit society, include such factors as increasing prosocial behaviour and reducing mental health issues, with the results cascading into lower health care costs.

The likelihood of this benefit occurring is ‘likely’.

The benefit score for Scenario 5 is 4.0×5.0=20.0 (high).

The highest benefit score of 20.0 (high) occurred for Scenario 5.

#### 4.1.3. BRA Evaluation

**Figure 6:**
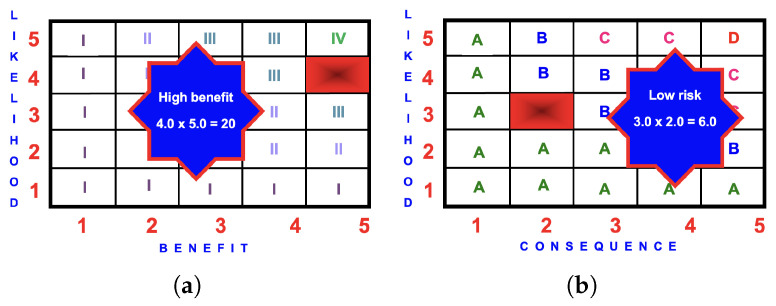
BRA matrices for children participating in R&T play within a supervised early childhood setting: (**a**) benefit score of 5.0×4.0=20.0 (high) or 4.0×5.0=20.0 (high); (**b**) risk score of 3.0×2.0=6.0 (low).

Figure 6 presents the BRA matrices for children participating in R&T play within a supervised early childhood setting. The benefit of 20.0 is greater than the risk of 6.0 and children engaging in R&T play should be encouraged as the benefit grossly exceeds the risk.

The benefits of playful aggression in the context of R&T play, especially in supporting the development of emotional and behavioural regulation, far outweigh any assumed consequences or unintended interactions.

### 4.2. Children Participating in a Nature Play Activity (NPA)

Learning environments that include natural elements have become growing features of play spaces in recent years. Natural elements such as grouping logs and tree stumps of varying heights and diameters into a cluster provide a more challenging and dynamic climbing experience for children than that afforded by standard, manufactured climbing apparatus. As a form of risky play (play with heights), this activity presents an experience for children with a range of benefits including:

- Creativity and problem solving;
- Coordination;
- Proprioception;
- Balance and agility;
- Gross motor skill development;
- Physical activity;
- Confidence building;
- Environment stewardship.

#### 4.2.1. Risk Matrix

The likelihood of an injury occurring from a child climbing/scrambling over a cluster of logs/tree stumps is ‘conceivable’ (2.0). The consequences are somewhere between ‘little or no injury’ and ‘minor injury requiring first aid’ (1.5).

The risk score is 2.0 × 1.5 = 3.0 (low).

#### 4.2.2. Benefit Matrix

The likelihood of children experiencing benefits from this activity is ‘certain’ (5.0). The highest benefit score occurs somewhere between Scenarios 4 and 5 when the likelihood is 5.0.

The benefit score is 5.0 × 4.5 = 22.5 (highest benefit).

#### 4.2.3. BRA Evaluation

**Figure 7:**
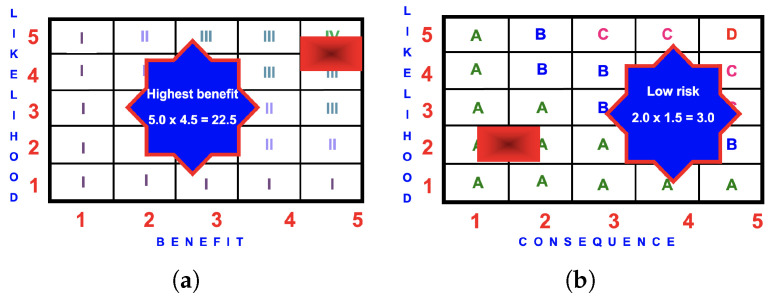
BRA matrices for children participating in an NPA: (**a**) benefit score of 5.0×4.5=22.5 (highest); (**b**) risk score of 2.0×1.5=3.0 (low).

Figure 7 presents the BRA matrices for children participating in an NPA.

### 4.3. Child Using a Swing in an Outdoor Playground (SiOP)

This example illustrates an example of a single-user play activity which provides children with a range of benefits including:

- Skill mastery;
- Reflection time;
- Coordination;
- Balance and vestibular stimulation;
- Swinging motion;
- Upper body and core strength;
- Exercise and lowering obesity levels;
- Sharing.

#### 4.3.1. Risk Matrix

The magnitude of the likelihood that an injury occurs when a child uses an SiOP will depend largely on the playground design. Was the playground designed to discourage children running in front of the moving swing by situating the swing unit outside the circulation space? Does the playground have an impact attenuating surface beneath the swing to protect the child from falling from the potential free height of fall? Do any of the chains and connectors introduce finger entrapment hazards? Does the leading edge of the swing seat have an impact attenuating surface? If the swing was installed and maintained to the relevant standards such as EN 1176-2:2017 Playground equipment and surfacing Part 2: Specific safety requirements and test methods for swings [54], none of these hazards would be present. If they did exist, they would need to be eliminated or mitigated. Incidents which can occur on a swing unit that complies with EN 1176-2:2017 include falls from the swing seat while the child is dismounting and children running in front of the moving swing.

Likelihood =3.0 (possible).

The magnitude of the consequence if an injury with a likelihood of 3.0 does occur from a child being impacted by the moving swing would be a minor injury requiring first aid.

Consequence =2.5 (minor to moderate injury)

The risk score is 3.0×2.5=7.5 (medium).

#### 4.3.2. Benefit Matrix

Again, there are various different combinations of likelihood and benefit for this example. Five combinations for a child using a swing will be discussed.

Scenario 1
  A momentary benefit, such as joy and exhilaration emanating from the activity. The likelihood of this benefit occurring is ‘almost certain’.

The benefit score for Scenario 1 is 5.0×1.0=5.0 (low).

Scenario 2
  Short-term benefit, such as having learned a new skill or learning skills faster. The likelihood of this benefit occurring is ‘certain’.

The benefit score for Scenario 2 is 5.0×2.0=10.0 (medium).

Scenario 3
  Medium-term benefit, such as gaining proficiency that opens new opportunities and beginnings of a benefit feedback loop. The likelihood of this benefit occurring is ‘certain’.

The benefit score for Scenario 3 is 5.0×3.0=15.0 (high).

Scenario 4
  Permanent or lifelong lifestyle improvements are a necessary precursor to improved physical, social, and mental health and well-being outcomes. It can also have a positive influence on future engagement levels that cascade into further benefits for the end-user and the likely permanence of the benefit feedback loop. This can also encourage and reinforce the user’s engagement in greater challenges. The likelihood of this benefit occurring is ‘certain’.

The benefit score for Scenario 4 is 5.0×4.0=20.0 (high).

Scenario 5
  Benefits that go beyond the individual to engage others and potentially benefit society, such as a reduction in suicide rates because of the lowering of depression, and the ripple effect results, e.g., lowering health care costs. The likelihood of this benefit occurring is ‘likely’.

The benefit score for Scenario 5 is 4.0×5.0=20.0 (high).

The highest benefit score of 20.0 (high) occurred for Scenarios 4 and 5.

#### 4.3.3. BRA Evaluation

**Figure 8:**
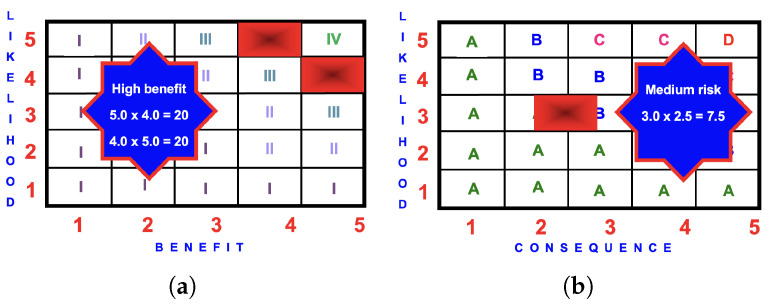
BRA matrices for a child using an SiOP: (**a**) benefit score of 5.0×4.0=20.0 (high) or 4.0×5.0=20.0 (high); (**b**) risk score of 3.0×2.5=7.5 (medium).

Figure 8 presents the BRA for a child using an SiOP. The benefit of 20.0 is greater than the risk of 7.5 and riding a swing should be encouraged as the benefit grossly exceeds the risk.

### 4.4. A Child Riding a Flying Fox in an Outdoor Playground (FFiOP)

The FFiOP example illustrates an instance of single-user play activity which provides children with a range of benefits including:

- Skill mastery;
- Upper body strength (for hanging flying fox);
- Exercise (dragging fox to dispatch);
- Excitement and fear (acceleration and deceleration forces);
- Gliding motion;
- Sharing.

#### 4.4.1. Risk Matrix

The magnitude of the likelihood that an injury occurs when a child rides an FFiOP will depend to a large extent on the familiarity the child has with the device. This is a skill-based activity which means a first-time or novice user is more likely to suffer an injury than a child who is familiar with the device [55]. The correct setup and maintenance will reduce the likelihood and degree of injuries occurring [45,56,57]. Is the stopper in the correct position? Is the flying fox designed to slow the user gradually or does it stop them abruptly? Is the maximum speed limited to less than 7 m/s? Is there minimum 400 mm seated user clearance along the entire length of the main cable? Is the free height of fall less than the maximum allowable, i.e., 1.5 m seated or 2.1 m hanging? The degree to which each particular flying fox complies with these safety requirements will influence the likelihood and consequences of an incident occurring. The possibility of an incident while a child is using a flying fox is somewhere between ‘could occur’ and ‘almost certain’.

Likelihood =3.5 (possible to likely).

The magnitude of the consequence if an injury does occur from a child riding an FFiOP when the likelihood is 3.5 is a minor injury requiring first aid.

Consequence =2.0 (minor injury requiring first aid)

The risk score is 3.5×2.0=7.0 (medium).

#### 4.4.2. Benefit Matrix

The highest benefit score occurs somewhere between Scenarios 3 and 4 when the likelihood is 4.5.

The benefit score is 3.5×4.5=15.8 (high).

#### 4.4.3. BRA Evaluation

**Figure 9:**
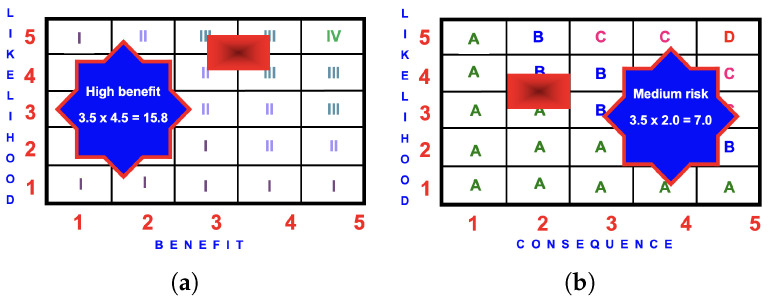
BRA matrices for an FFiOP: (**a**) benefit score of 3.5×4.5=15.8 (high); (**b**) risk score of 3.5×2.0=7.0 (medium).

Figure 9 is an example of the BRA for a child riding an FFiOP.

The benefit of 15.8 is greater than the risk of 7.5 and the flying fox should be encouraged, as the benefit exceeds the risk. The risk associated with this activity is maintenance sensitive, so it is important for the owner/operator to ensure the flying fox is regularly inspected and maintained to retain the benefit of this play activity.

### 4.5. A Child on a Rocking Device in an Outdoor Playground (RDiOP)

The RDiOP example illustrates a case of a single-user play activity which is normally installed in a cluster, so that two or more children can interact with each other while playing. The provision of these devices has a range of inherent benefits for children, including:

- Skill mastery;
- Coordination;
- Balance;
- Rocking motion and the development of their vestibular system;
- Upper body and core strength;
- Exercise and physical exertion;
- Sharing and taking turns;
- Gross motor skill development.

#### 4.5.1. Risk Matrix

The likelihood of an injury occurring from a child riding an RDiOP is somewhere between ‘highly unlikely’ to ‘conceivable’ (1.5). The consequences are somewhere between ‘little or no injury’ and ‘minor injury requiring first aid’ (1.5).

The risk score is 1.5×1.5=2.3 (low).

#### 4.5.2. Benefit Matrix

The highest benefit score occurs somewhere between Scenarios 3 and 4 when the likelihood is 4.5.

The benefit score is 3.5×4.5=15.8 (high).

#### 4.5.3. BRA Evaluation

**Figure 10:**
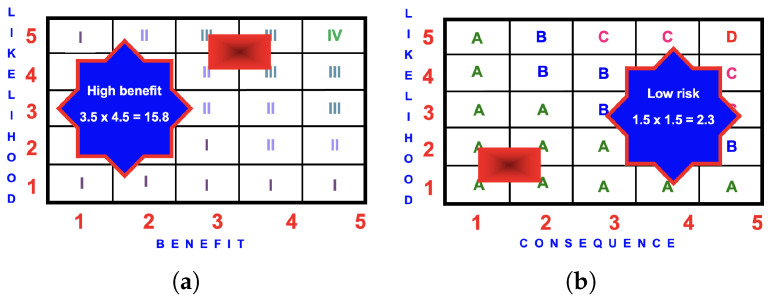
BRA matrices for a child on an RDiOP: (**a**) benefit score of 3.5×4.5=15.8 (high); (**b**) risk score of 1.5×1.5=2.3 (low).

Figure 10 is an example of the BRA for a child on an RDiOP.

The benefit of 15.8 is greater than the risk of 7.0 and the activity of small children riding rocking devices should be encouraged as the benefit exceeds the risk.

### 4.6. A Group of Children Climbing up and Playing on a 10 m High 3D Spatial Net (3DSN)

The 3DSN example illustrates an example of a multiuser play activity which provides a large number of children with a range of benefits including:

- Skill mastery;
- Coordination;
- Climbing skills;
- Balance and agility;
- Upper body strength;
- Exercise and physical activity;
- Reduced fear of heights;
- Building confidence and independence;
- Gross motor skill development.

As stated earlier, play (especially risky play) is a crucial element in a child’s life to develop the prerequisite dispositions and foundational skills for later in life. A poignant example is a 10 m high 3D spatial net which allows children of aged 6–18 to play together and self-calibrate their personal exposure to risk. They will climb only as high as they dare and if in the unlikely event that they fall, the space net is designed to separate this fall from a height of more than 3 m height through a series of lesser height falls as they descend like a rag doll through a non-rigid spatial net structure.

#### 4.6.1. Risk Matrix

The likelihood of an injury occurring from a group of children climbing up and playing on a 10 m high 3D spatial net in an outdoor playground is ‘possible’ (3.0). The consequences are somewhere between ‘minor injury requiring first aid’ (2.0).

The risk score is 3.0×2.0=6.0 (low).

#### 4.6.2. Benefit Matrix

The highest benefit occurring for a group of children climbing up and playing on a 10 m high 3DSN occurs somewhere between Scenarios 3 and 4 when the likelihood is 4.5.

The benefit score is 3.5×4.5=15.8 (high).

#### 4.6.3. BRA Evaluation

**Figure 11:**
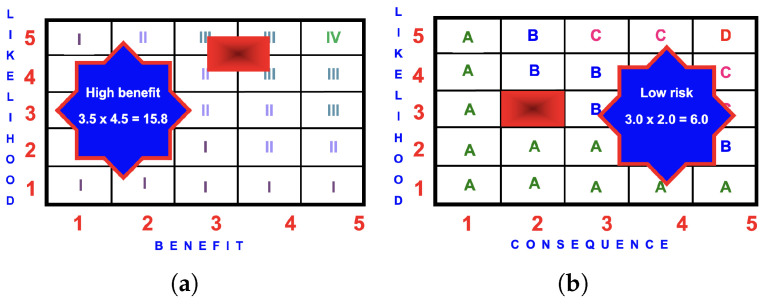
BRA matrices for a 3DSN: (**a**) benefit score of 3.5×4.5=15.8 (high); (**b**) risk score of 3.0×2.0=6.0 (low).

The benefit of 15.8 is greater than the risk of 7.0 and the 10 m high 3DSN should be encouraged as the benefit exceeds the risk. Figure 11 is an example of the BRA for a group of children climbing and playing on a 10 m high 3DSN.

### 4.7. Giant Tube Slide Being Used Inappropriately and Appropriately

This example illustrates two instances of a single-user play activity. For the first example, there is no benefit, whereas in the second example, the benefit is greater than the risks.

The benefits in this play activity include the following:

- Skill mastery;
- Coordination;
- Exercise (climbing to the start);
- Builds confidence and independence;
- Fosters risk tolerance;
- Gross motor skill development.

In recent years, giant tube slides have been installed within children’s playgrounds. The discussion around these devices is divided. On the one hand, they are exciting and provide the older and more risk-taking user with an appropriate and stimulating play activity. On the other hand, they introduce a level of risk which can be inappropriate for younger children, or children and their carers riding in tandem.

#### 4.7.1. Inappropriate and Hazardous Use of a Giant Tube Slide (GTSi)

This example illustrates a hazardous situation where the risk outweighs benefit and risk elimination or mitigation is required. For the younger inexperienced child, or a small child and their carer (where sliding is done in tandem), these large tube slides can be extremely hazardous. These playground users have undoubtedly observed experienced users enjoying themselves and sliding without incident. This observation offers a false sense of security. The small child wants to ride the slide so the parent wishing to please their child places them in their lap.

However, the giant slide is a device which requires a degree of skill and knowledge to ride. For example, when ridden for the first time, the user’s experience is faster and more dynamic than anticipated. From their launch, their speed increases rapidly and the journey along the tube is essentially unknown. These factors result in the user panicking, and usually attempting to stop the momentum.

There are two common outcomes for the younger inexperienced child, or a small child and their carer:

1. The parent who has less control (as they are holding their child between their legs) panics when their velocity increases above their perceived ‘safe’ threshold. The adult attempts to slow down and in so doing loses control and suffers a serious injury, such as a broken ankle, tibia, or femur.
2. The small child or inexperienced rider uses the slide. In a similar manner to the parent and child, their velocity increases above their perceived ‘safe’ threshold. The child attempts to slow down and in so doing suffers a similar fate to the adult travelling in tandem with a child between their legs.

#### 4.7.2. Risk Matrix

The likelihood of an injury occurring from using a hazardous slide is ‘certain’ (5.0).

The consequences are ‘serious injury with long-term consequences’ (4.0).

The risk score is 5.0×4.0=20.0 (high).

#### 4.7.3. Benefit Matrix

There is no benefit to this activity for the younger child or the tandem giant tube slide users.

#### 4.7.4. BRA Evaluation

The risk is high and there is no benefit to this activity, and therefore, it should not proceed.

#### 4.7.5. Appropriate and Beneficial Use of a Giant Tube Slide (GTSa)

This example illustrates a situation where the giant tube slide is designed and installed by a competent manufacturer/supplier and the benefits slightly outweigh benefit risks. As a minimum, all slides must be designed, certified, manufactured, installed, maintained, and inspected to EN 1176-3:2017 [58]. In response to an increase in frequency and severity of giant tube slide incidents, some countries, such as Australia, have introduced additional guidelines for giant tube slides with the publication of SA HB 244:2025 Supplementary guide to AS 4685.3:2021 – Giant tube slides [59] (SA HB 244:2025). Design interventions contained within SA HB 244:2025 include the following:

- Centreline radius of all slide elbows to be greater than 1.2 m;
- Ability filters to limit access to the starting section of the slide;
- Anticlimb barriers to deter climbing on the external parts of the slide.

**Figure 12:**
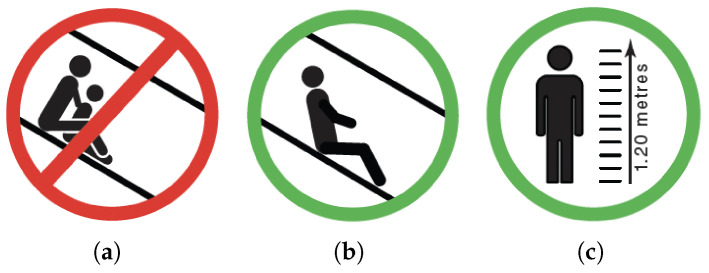
Example of suggested signage for giant tube slides: (**a**) no tandem riding; (**b**) feet first; (**c**) users taller than 1.2 m.

It is recommended that all giant tube slides that do not include these interventions as a minimum be removed or modified. In addition to the design intervention, appropriate signage is also recommended. Figure 12 provides examples of recommended signage [59]:

- Advice against tandem riding;
- Instructions to slide feet first only;
- Warn against slowing or stopping inside the slide;
- All users to be 1.2 m or taller.

#### 4.7.6. Risk Matrix

The likelihood of an injury occurring from using a hazardous slide is ‘possible’ (3.0).

The consequences are ‘moderate injury requiring medical assessment’ (3.0).

The risk score is 3.0×3.0=9.0 (medium).

#### 4.7.7. Benefit Matrix

The highest benefit occurring for the giant tube slide occurs Scenario 3 when the likelihood between ‘likely’ 4.0 and ‘certain’ 5.0.

The benefit score is 4.5×3.0=13.5 (medium).

#### 4.7.8. BRA Evaluation

The benefit score of 13.5 is greater than the risk score of 9.0 and the activity should proceed. The perception of the giant slide is that this is a ‘risky’ activity. However, when it is accessed, the benefit of a well-designed giant slide outweighs these negative perceptions.

**Figure 13:**
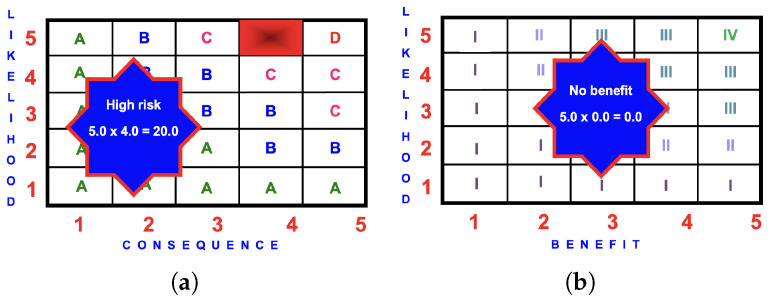
BRA matrices for a GTSi: (**a**) risk score of 5.0×4.0=20.0 (high); (**b**) benefit score of 0.0 (no benefit).

**Figure 14:**
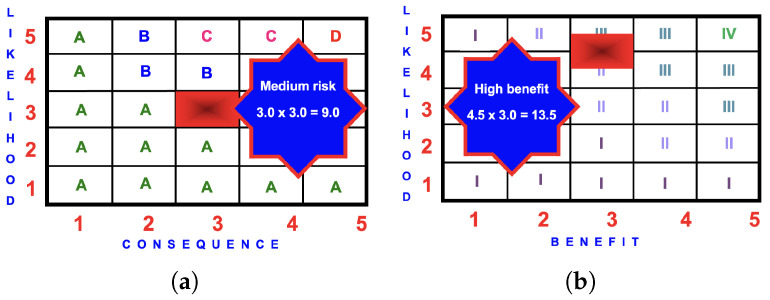
BRA matrices for a GTSa: (**a**) risk score of 3.0×3.0=9.0 (medium); (**b**) benefit score of 4.5×3.0=13.5 (high).

Figure 13 and Figure 14 present a comparison between a poorly designed and hazardous giant tube slide and a well designed beneficial giant tube slide.

### 4.8. Broken Glass Bottle in the Outdoor Playground or School Setting (BGB)

This example illustrates a hazardous situation where there is no benefit and where risk elimination is warranted.

#### 4.8.1. Risk Matrix

The magnitude of the likelihood of an injury occurring from the broken glass bottle will primarily depend on where the broken glass is located. If it is assumed that the broken glass is close to the playground equipment, it is likely or almost certain that a child will be injured. If the playground is busy, there will be more children playing and the likelihood will be increased.

Likelihood =5.0 (almost certain)

The consequence of an injury from a broken glass in an outdoor playground will depend, for the most part, on how the injury occurs. A child could sever an artery, a small child could swallow the shards of glass, they could tread on the glass with bare feet, or they could injure themselves in a myriad of other ways. Each particular likelihood needs to be linked with the associated consequence with the highest consequence. This may or may not be the scenario with the highest risk score.

The consequence which is linked to ‘almost certain’ is a likelihood somewhere between moderate and serious.

Consequence =3.5 (moderate/serious).

The risk score is 5.0×3.5=17.5 (high).

Alternatively, the consequence of a severed artery which is a ‘serious injury’ would be linked to a likelihood of ‘possible’.

The risk score is 3.0×4.0=12.0 (medium).

This same process could be conducted for every combination of likelihood and consequence and the one with the greatest risk score is chosen. For the broken glass bottle the highest risk score is 17.5 (high).

#### 4.8.2. BRA Evaluation

**Figure 15:**
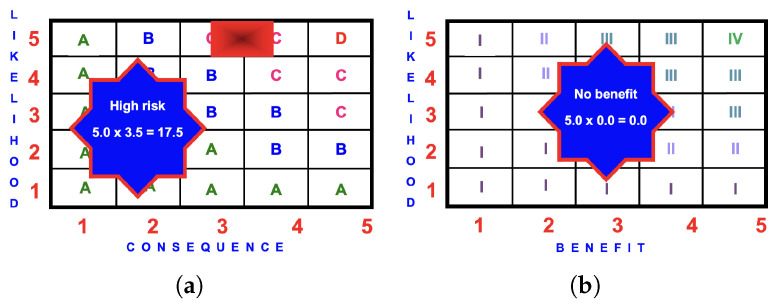
BRA matrices for the BGB: (**a**) risk score of 5.0×3.5=17.5 (high); (**b**) benefit score 0.0 (no benefit).

The risk grossly outweighs the zero benefit and the broken glass bottle ought to be removed from the playground as soon as is reasonably practicable. Figure 15 is an example of the BRA for the BGB.

### 4.9. Comparison of the Examples

Table 1 is a compilation the data contained within each of the examples above.

### 4.10. Limitations

The researchers are mindful that further studies are needed to ascertain if the scores generated by the BRA tool are reliable across users, and whether they lead to sound and consistent decision making in practice. To this end, the authors are cognisant that the quantification should be both purposeful and accurate, with distinct evidence accompanying the generalisability of the descriptors and scores. These variations will be addressed in future publications.

Ostensibly, the validation of the BRA instrument is contingent upon evaluating the central claims, assumptions, and inferences that connect assessment scores, with their intended interpretations and uses [60]. Lastly, Hess and Kvern [61] stated that Kane’s frame for validation supports the interpretation and use of assessment scores, assuming they are valid and appropriate for the intended purpose.

## 5. Conclusions

Although risk has many inherent benefits, it is vital to balance risk taking with injury prevention in the play environment. The additional information offered by the BRA introduced in this paper enables a more realistic and comprehensive calculation than the traditional methods relied on to date, such as those contained with IEC 31010:2019 [46]. The proposed tool uses two separate matrices, namely a benefit matrix and a risk matrix, to quantify and allow comparison of both the positive and negative attributes of each individual situation within outdoor play activities and outdoor learning environments such as playgrounds.

It is undisputed that hazardous situations in playgrounds have no benefit and should be eliminated or mitigated to a tolerable level if they cannot be removed. However, where the BRA can measure and demonstrate a net positive impact in the context of risk measurement, children can and should enjoy an educative and more rewarding play experience. Playground inspectors around the world are encouraged to use this BRA in their assessments within outdoor play activities and outdoor learning environments such as playgrounds. Holistically, if we want to enhance the cognitive and developmental growth of our children, the provision of measured risk taking is warranted. Initiative skills, executive function, self-regulation, and self-confidence are just a few of the lifelong benefits that are accrued from managed risk exposure.

If society can make playgrounds and outdoor learning activities exciting, enjoyable, and stimulating, children will be naturally attracted to these places and spaces. Benefits that go beyond the individual-centric approach to engage more broadly with others will benefit society in a myriad of ways. This includes problem solving skill development, prevention of overweight, improved mental health, and the reinforcement of enjoying life-long physical exercise, among many more benefits. Ultimately, these outcomes lower the burden of disability and associated health care costs and increase the GDP of the nation.

Lastly, there are a plethora of opportunities to conduct future research in the fields of outdoor pursuits, adventure education, and ‘risky’ sporting activities, to name just a few. In short, the BRA tool has utility a wide range of leisure, recreation, and sporting pursuits. These opportunities will enable it to be tested for assessing benefit–risk in other specialised domains.

## Figures and Tables

**Table 1:** Compilation of the data contained within the examples.

	Benefit	Benefit	Benefit	Risk	Risk	Risk	BRA
	Like’d	Conseq.	Score	Like’d	Conseq.	Score	B > R
R&T	5.0	4.0	III (20.0)	3.0	2.0	A (6.0)	Yes
NPA	5.0	4.5	IV (22.5)	2.0	1.5	A (3.0)	Yes
SiOP	5.0	4.0	III (20.0)	3.0	2.5	B (7.5)	Yes
FFiOP	3.5	4.5	III (15.8)	3.5	2.0	A (7.0)	Yes
RDiOP	3.5	4.5	III (15.8)	1.5	1.5	A (2.3)	Yes
3DSN	3.5	4.5	III (15.8)	3.0	2.0	A (6.0)	Yes
GTSi	–	–	– (0.0)	5.0	4.0	C (20.0)	No
GTSa	4.5	3.0	III (13.5)	3.0	3.0	A (3.0)	Yes
BGB	–	–	– (0.0)	5.0	3.5	C (17.5)	No

## Data Availability

The original contributions presented in this study are included in the article. Further inquiries can be directed to the corresponding author.

## Abbreviations

The following abbreviations are used in this manuscript:

AS 4685.0:2017	AS 4685.0:2017 Playground equipment and surfacing Part 0:
	Development, installation, inspection, maintenance and operation
AS 4685.1:2004	AS 4685.1:2004 Playground equipment and surfacing Part 1:
	General safety requirements and test methods
BRA	Benefit–risk analysis
IEC 31010:2019	IEC 31010:2019 Risk management – Risk assessment techniques
ISO 4980:2023	ISO 4980:2023 Benefit–risk assessment for sport and recreational
	facilities, activities and equipment
EN 1176-1:2008	EN 1176-1:2008 Playground equipment and surfacing Part 1:
	General safety requirements and test methods
EN 1176-2:2017	EN 1176-2:2017 Playground equipment and surfacing Part 2:
	Specific safety requirements and test methods for swings
Play Safety Forum	Play Safety Forum, Managing risk in play provision: A position statement
SA HB 244:2025	SA HB 244:2025 Supplementary guide to AS 4685.3:2021 – Giant tube slides
